# miR-145 expression enhances integrin expression in SK-GT-4 cell line by down-regulating c-Myc expression

**DOI:** 10.18632/oncotarget.24613

**Published:** 2018-03-08

**Authors:** Mathieu Francois Derouet, Eugenia Dakpo, Licun Wu, Guan Zehong, James Conner, Shaf Keshavjee, Marc de Perrot, Thomas Waddell, Elena Elimova, Jonathan Yeung, Gail Elizabeth Darling

**Affiliations:** ^1^ Latner Thoracic Surgery Research Laboratories, Princess Margaret Cancer Research Tower, University Health Network, Toronto, Ontario, Canada; ^2^ Department of Surgery, Division of Thoracic Surgery, Toronto General Hospital, University Health Network, Toronto, Ontario, Canada; ^3^ Department of Medical Oncology and Hematology, Princess Margaret Cancer Centre, University Health Network, Toronto, Ontario, Canada; ^4^ Department of Pathology and Laboratory Medicine, Mount Sinai Hospital, Toronto, Ontario, Canada

**Keywords:** esophageal adenocarcinoma, miR-145, c-Myc, integrins, metastasis

## Abstract

Adenocarcinoma of the esophagus is increasing in frequency and is the 6th most common cause of cancer death in North America. In adenocarcinoma cell lines, we have previously demonstrated that expression of miR-145, leads to enhanced invasion, resistance to anoikis and better attachment to fibronectin in esophageal adenocarcinoma. In contrast, expression of miR-145 acts as a tumor suppressor in squamous cell carcinoma. The molecular mechanisms responsible for the oncogenic effects of miR-145 were investigated. In this report, we demonstrate that we can partially recreate the miR-145 effects in EAC by knock down of the expression of c-Myc, which is one of the targets of miR-145. Knocking down of c-Myc expression resulted in upregulation of integrin subunits α5 and β3. Finally, we demonstrated that integrin α5 expression correlates to fibronectin attachment potential whereas integrin β3 expression correlates with resistance to anoikis and invasion potential. Finally, we demonstrate that expression of miR-145 in esophageal adenocarcinoma cell line (SK-GT-4) enhances tumor growth and metastasis in a NOD/SCID xenograft model. Overall, the oncogenic potential of miR-145 in EAC appears to be mediated by downregulation of c-Myc leading to the expression of integrins subunits α5 and β3.

## INTRODUCTION

Carcinoma of the esophagus has a high case fatality ratio. Over the past 20 years, the incidence of the esophageal adenocarcinoma (EAC) subtype has been increasing in North America and Europe [1]. Esophageal cancer has now become the eighth most common cancer and the sixth most common cause of cancer death in men [2]. This dramatic increase has been associated with gastroesophageal reflux disease (GERD), obesity and Barrett's esophagus, which increases the risk of esophageal adenocarcinoma by 30-fold [3]. Recent literature has highlighted the role of microRNA (miRNA) in cancer progression and chemotherapy resistance. miRNAs have been shown to play an important role in the regulation of cell differentiation, proliferation and apoptosis [4–7]. As deregulation of these processes are features of cancer, it is likely that miRNAs play a role in carcinogenesis. Previous studies have reported that the expression of miRNAs is altered in cancers, linking miRNA expression to either initiation or progression of cancers such as breast, lung, pancreas, prostate and CLL [8].

We previously reported on the miRNA profiles of esophageal cancer before and after neoadjuvant therapy [9]. We found 568 miRNAs, which were significantly up or down-regulated after neoadjuvant therapy. We also established that post-treatment high levels of miR-135b and miR-145 (which were induced by neoadjuvant therapy) were linked to a shorter disease-free survival. miR-135b has been described in the literature as a tumor promoter. It plays a central role in colorectal cancer progression and promotes metastasis in lung cancer [10–11]. However, miR-145 has been described as a tumor suppressor miRNA in a variety of cancers such as breast, lung, colon and stomach [12]. In esophageal squamous cell carcinoma (ESCC), miR-145 expression is downregulated; when it is expressed, it inhibits cell proliferation and cell invasion [13–14]. Surprisingly, in EAC, we showed that miR-145 was oncogenic and was able to protect the cells against cell death induced by detachment (anoikis) and enhance cell invasion [15]. This report illustrated the dual action of miR-145 in the esophagus.

In this report, the signaling pathways responsible for the oncogenic effects of miR-145 in EAC leading to the enhancement of the metastatic process are investigated.

## RESULTS

### miR-145 expression upregulates integrin expression in SK-GT-4

**Figure 1:**
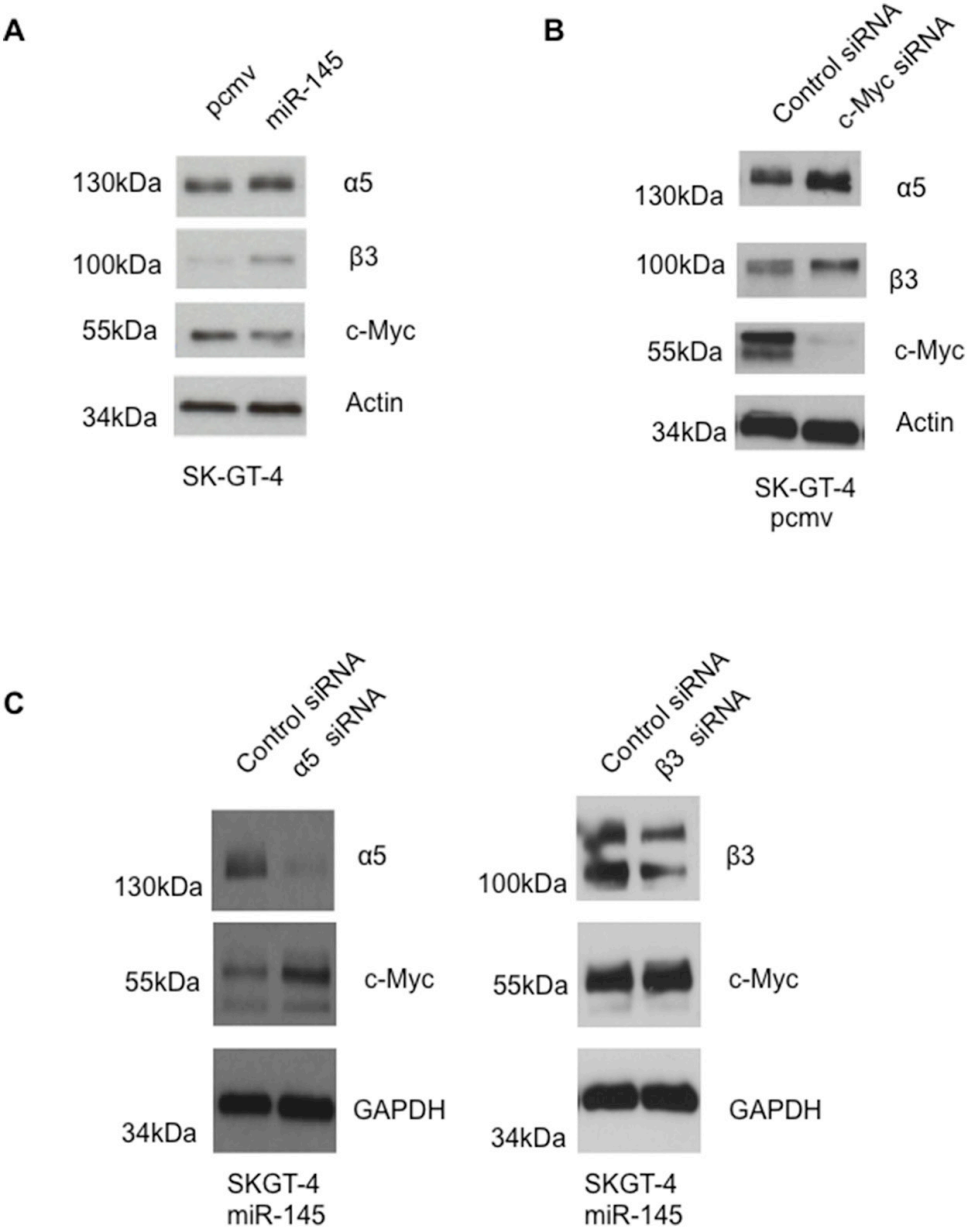
miR-145 expression in EAC cells leads to higher expression of integrins α5 and β3 but lower expression of c-Myc **(A)** Western Blot looking at the expression of integrins α5, β3 and c-Myc in SK-GT-4 pcmv and miR-145, n=3. **(B)** Western Blot analysis of integrins α5, β3 and c-Myc after siRNA transfection in SK-GT-4 pcmv cells, n=3. **(C)** Western Blot analysis of integrins α5, β3 and c-Myc after siRNA transfection in SK-GT-4 miR-145 cells, n=3.

We previously reported that miR-145 expression in EAC cell lines enhanced anoikis resistance and invasion potential [15]. We decided to study the signaling pathways required for those effects. In our previous manuscript, we showed that binding to fibronectin was enhanced in the miR-145 expressing cells. As adhesion to fibronectin is mediated through integrins, specifically α5β1 (also known as the fibronectin receptor) and αvβ3, integrin expression was investigated. Integrin subunits were measured using Western Blotting. There was no difference in levels of β1, αv and β4 expression between pcmv and miR-145 cells (Supplementary Figure 1C and Supplementary Figure 1D). However, miR-145 cells expressed higher levels of integrin α5 and β3 subunits compared to the pcmv cells (Figure 1A). Similar results were observed in the EAC cell line OE33, where miR-145 cells expressed higher levels of integrin α5 but not of integrin β3 subunits (Supplementary Figure 1A).

### c-Myc is a repressor of integrin α5 and β3 subunits in SK-GT-4 cells

As miRNAs can only downregulate expressions of their targets, the only way miRNAs can upregulate protein expression is by downregulation of a repressor of the protein. Therefore, to investigate the pathway by which miR-145 leads to upregulation of integrin α5 and β3, the role of the oncogene c-Myc was evaluated. c-Myc is a well known target of miR-145 and has been shown, in the literature, to repress the expression of several integrins in cancer cells [12, 17]. Thus c-Myc is a potential link to explain the effects of miR-145 in SK-GT-4.

c-Myc expression was evaluated by Western Blotting. SK-GT-4 miR-145 cell lines expressed less c-Myc than their relative control (Figure 1A) whereas the SK-GT-4 miR-145 cell lines expressed higher levels of integrin α5 and αvβ3. When c-Myc was downregulated in the pcmv cells, an upregulation of integrin α5 and β3 was observed, similar to the level showed in the miR-145 cells (Figure 1B). No difference was observed in the integrin β1 level (Supplementary Figure 1D). The reverse of that effect was observed in the miR-145 cells transfected with integrin α5 and β3 siRNA (Figure 1C).

### c-Myc knock down results in increased cell adhesion to fibronectin and resistance to anoikis

**Figure 2:**
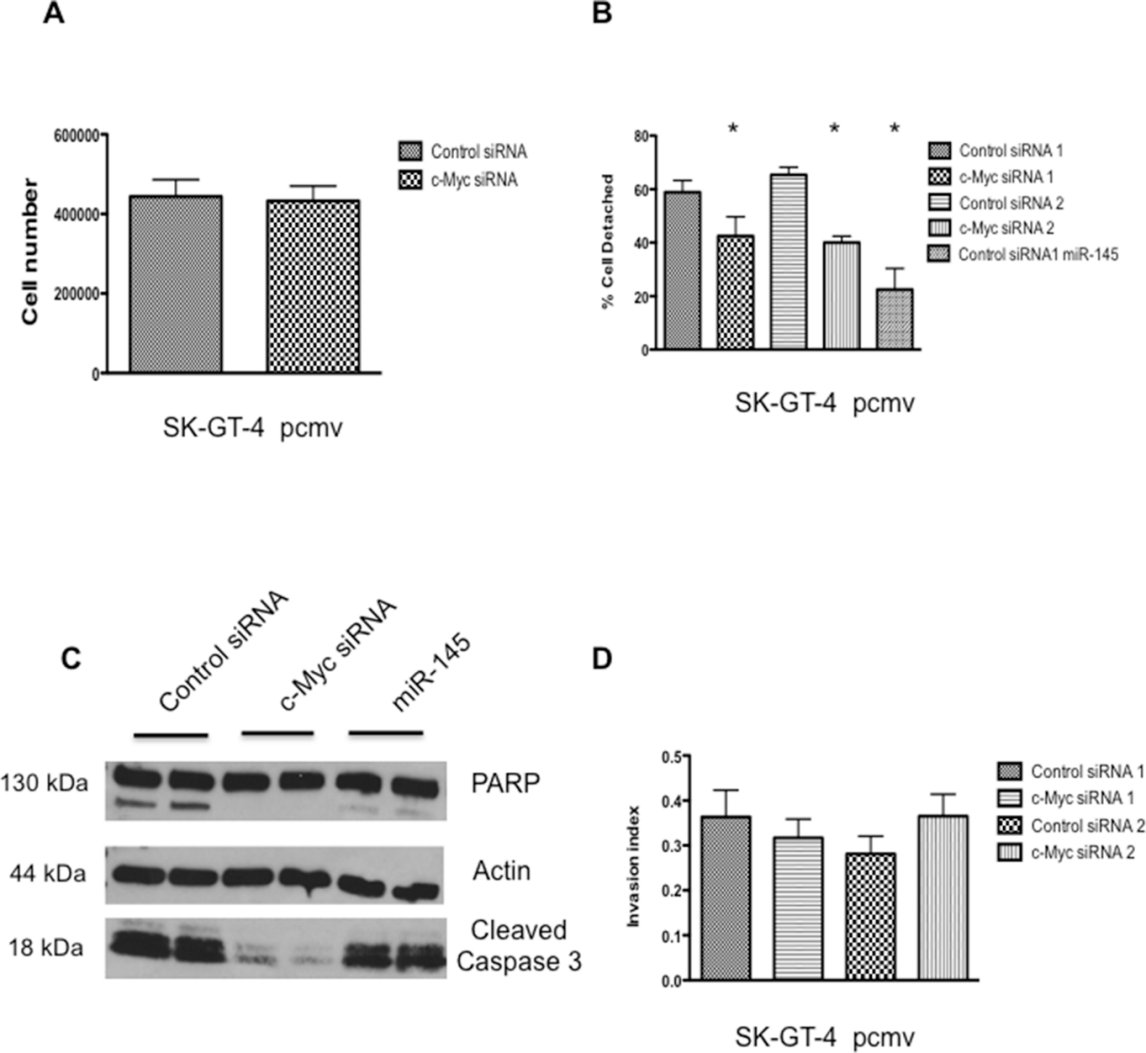
c-Myc knockdown partially mimics expression of miR-145 in SK-GT-4 cell line **(A)** Cell proliferation assay with SK-GT-4 pcmv after c-Myc siRNA transfection, n=3. **(B)** Fibronectin adhesion assay with SK-GT-4 pcmv after c-Myc siRNA transfection, n=3, ^*^: p<0.05. **(C)** Western blot analysis of an anoikis assay (72h) with SK-GT-4 pcmv after c-Myc siRNA transfection, n=3. **(D)** Invasion assay with SK-GT-4 pcmv after c-Myc siRNA transfection, n=3.

**Figure 3:**
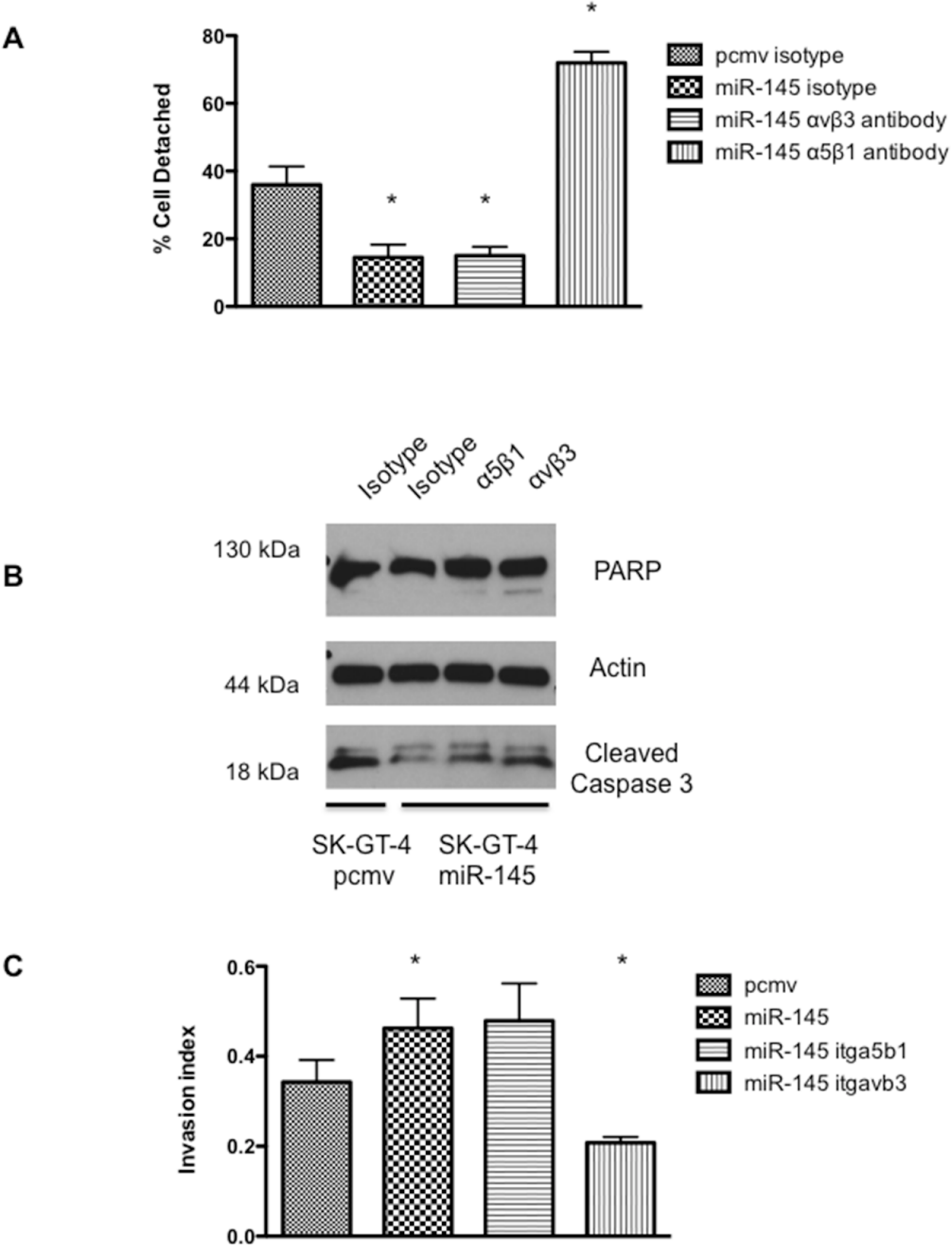
α5β1 inhibition reverts the effect of miR-145 on fibronectin adhesion whereas αvβ3 inhibition blocks the effect of miR-145 on anoikis resistance and cell invasion **(A)** Fibronectin Adhesion assay with SK-GT-4 miR-145. Cells were preincubated with either isotype or α5β1 or αvβ3 antagonist before plating on fibronectin coated plates, n=3, ^*^: p<0.05. **(B)** Western blot analysis of an anoikis assay (72h) with SK-GT-4 miR-145 cells preincubated with either isotype, α5β1 or αvβ3 antagonist antibody, n=3. **(C)** Invasion assay with SK-GT-4 miR-145 after incubation with either isotype, α5β1 or αvβ3 antagonist antibody, n=3, ^*^: p<0.05.

To determine if we could reproduce the effects of miR-145 on cell invasion and anoikis resistance results by downregulating c-Myc, siRNA transfection was used to down regulate expression of c-Myc in SK-GT-4 pcmv cells (Figure 1B). There was no difference in cell proliferation between the control siRNA and the c-Myc siRNA cells 72h after transfection (Figure 2B); however the c-Myc siRNA cells exhibited better adhesion to fibronectin coated plates compared to control siRNA (Figure 3B). After incubating the cells in suspension for 72h, c-Myc siRNA cells were able to survive better than the matching control as demonstrated by finding almost no cleaved PARP by Western blot (Figure 2C). Finally, on the cell invasion assay, surprisingly the c-Myc siRNA cells were invading at the same rate as the control cells (Figure 2D). By knocking down c-Myc expression in SK-GT-4 pcmv, we were able to recreate some of the results obtained with SK-GT-4 miR-145. This suggests that miR-145 oncogenic abilities seem to require c-Myc downregulation.

### Inhibition of the integrin α5β1 blocks the effects of miR-145 on fibronectin adhesion

It seems that the abilities of miR-145 expressing cells on anoikis and cell invasion could be replicated by knock down of c-Myc. Since c-Myc and the integrin α5 are linked, blocking the integrin α5β1 may reverse the effects of miR-145. When α5β1 activity was blocked by a monoclonal antibody, the fibronectin attachment was completely blocked in SK-GT-4 miR-145 cells (Figure 3A). This result was expected since integrin α5β1 is the main fibronectin receptor. However, inhibition of α5β1 activity did not sensitize SK-GT-4 miR-145 cells to anoikis (Figure 3B) or decrease their invasive ability (Figure 3C). These results suggest the miR-145 increased expression of integrin α5 is associated with increased ability to attach to fibronectin but not cell invasion or anoikis resistance.

### miR-145 upregulation of integrin αvβ3 controls cell invasion and anoikis resistance in SK-GT-4

Since blocking the activity of integrin α5β1 did not affect cell invasion or anoikis resistance, we decided to test if blocking the activity of integrin αvβ3 would affect those events. Despite being known as the vitronectin receptor, it has been reported that integrin αvβ3 could also bind to fibronectin [18]. Integrin αvβ3 activity was blocked with a monoclonal antibody on the SK-GT-4 cell lines but no differences in adhesion to fibronectin between the control and antagonist antibody were observed (Figure 3A). However, when anoikis resistance with the αvβ3 antibody was evaluated, we noticed that the αvβ3 antibody appears to sensitize the cells to anoikis. Increased PARP cleavage and stronger caspase 3 cleavage was identified when the SK-GT-4 miR-145 cells where treated with the αvβ3 antibody compared to the isotype (Figure 3B). Furthermore, the ability of the SK-GT-4 miR-145 cells to invade was partially reduced when the integrin αvβ3 was blocked (Figure 3C). Overall, blocking the activity of integrin αvβ3 in SK-GT-4 cells appears to reverse the enhancement in cell invasion and anoikis resistance provided by the miR-145 expression.

### miR-145 expression enhances tumorigenic abilities of SK-GT-4 cells in mice model

**Figure 4:**
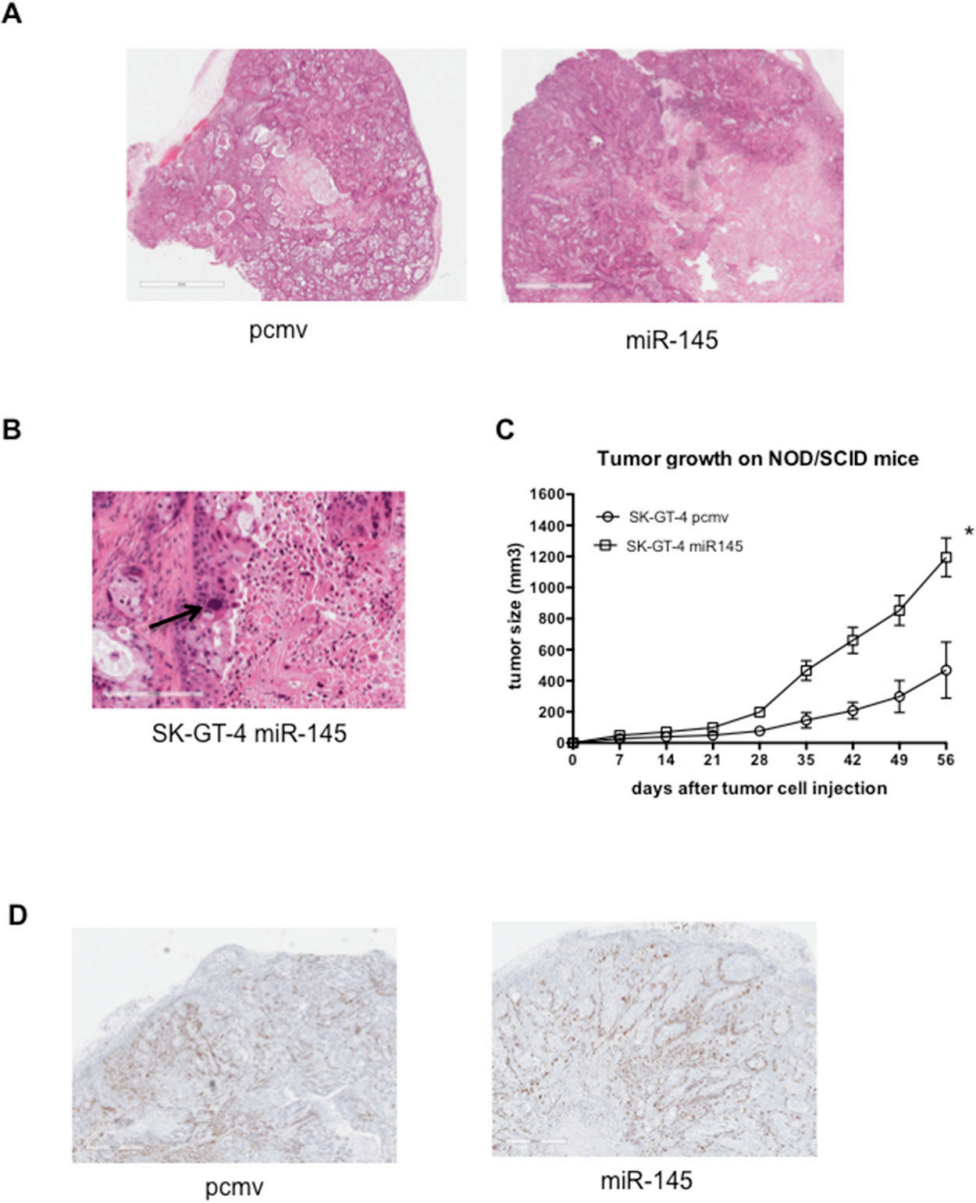
miR-145 expression in EAC cells leads to bigger tumors **(A)** Histology of the SK-GT-4 pcmv (left) and SK-GT-4 miR-145 (right) after 8 weeks. **(B)** Picture of a SK-GT-4 miR-145 tumor. The arrow points at the example of nuclear pleomorphism. **(C)** Growth curves of the SK-GT-4 tumors in NOD/SCID mice (n=9), ^*^: p<0.05. **(D)** Representative pictures of Ki67 staining of SK-GT-4 pcmv and miR-145 tumors.

Increased potential for invasion and resistance to anoikis as previously demonstrated in miR-145 overexpressing EAC cell lines (OE33 and SK-GT-4) could potentially result in more efficient metastasis. SK-GT-4 pcmv and miR-145 cells were injected into NOD/SCID mice). On histology, several differences were noted between the pcmv and miR-145 tumors. First, the miR-145 tumors displayed a greater geographic necrosis than the control tumors (all miR-145 tumors had necrosis compared to 50% of pcmv tumors). Necrotic areas represented 30%-40% of tumor volume for pcmv compared to 60%-70% of tumor volume in miR-145 tumors) (Figure 4A). This difference in necrosis is potentially due to the size of the tumors. Second, miR-145 tumors displayed a significantly greater cytologic atypia, approaching undifferentiated carcinoma in some areas. In the miR-145 tumors, atypical mitosis, marked nuclear pleomorphism (7-8x size ratios, Figure 4B) and bizarre nuclear shapes and contours were observed. These features are consistent with a more aggressive tumor, which is less likely to respond to chemotherapy treatment.

**Figure 5:**
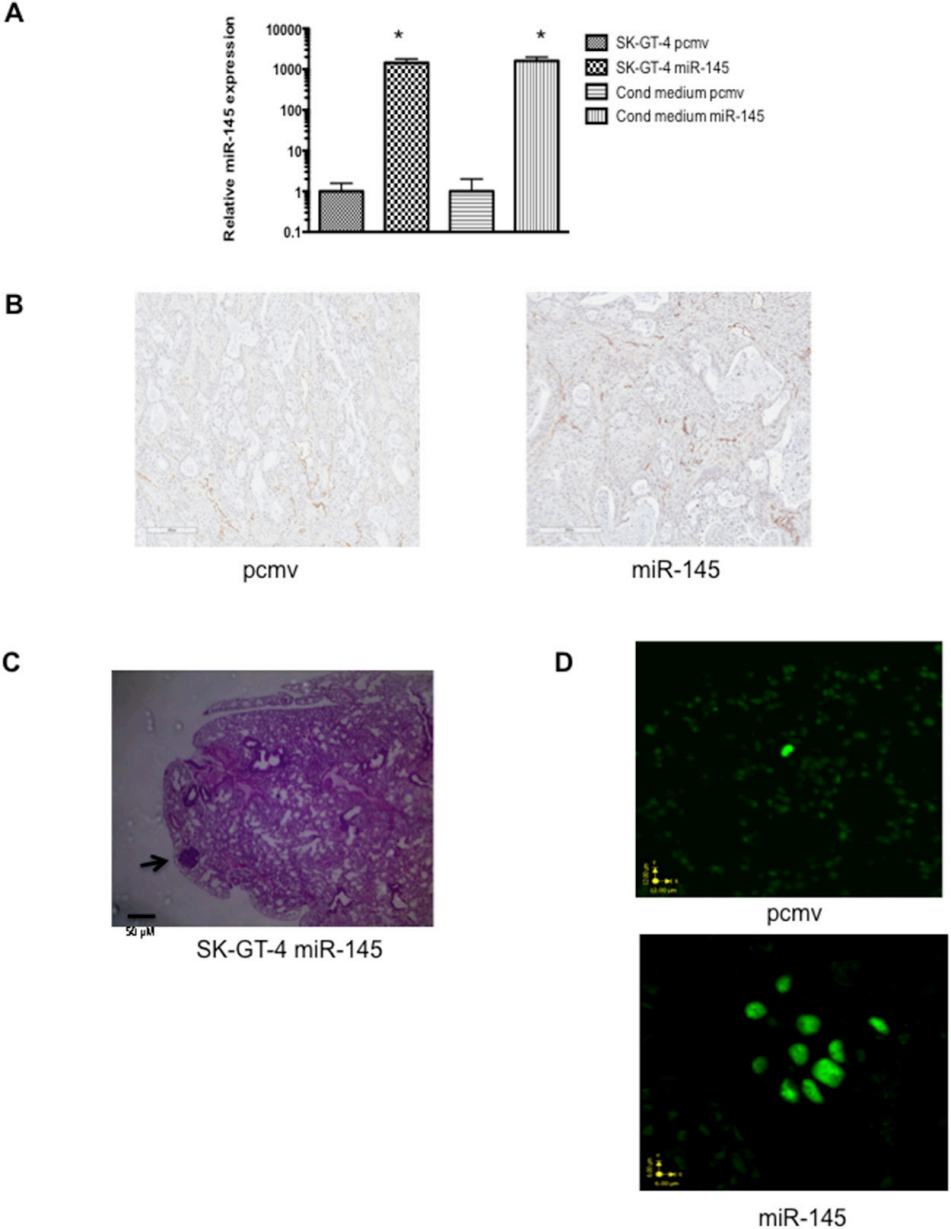
miR-145 expression in EAC increases metastasis but not angiogenesis in xenograft model **(A)** qRT-PCR results measuring miR-145 levels in SK-GT-4 pcmv and miR-145 cells as well as in their respective condition mediums, ^*^: p<0.05. **(B)** Representative pictures of CD31 staining of SK-GT-4 pcmv and miR-145 tumors. **(C)** Histology of the lung from a mouse injected with SK-GT-4 miR-145 cell line, the arrow points at the micro-metastasis. **(D)** Pictures of slides from lung of mice injected with SK-GT-4 pcmv (right) and miR-145 (left), stained with FISH and ALU sequence. The SK-GT-4 miR-145 shows more human cells in the lung as well as the beginning of formation of micrometastasis.

Around 4 weeks after flank injection of tumor cells, SK-GT-4 miR-145 cells formed larger tumors compared to pcmv (Figure 4A and Supplementary Figure 2A) and the miR-145 tumors were more than twice as big as the SK-GT-4 pcmv at the time of sacrifice. This was an unexpected result as cell proliferation between the two cell lines was previously evaluated and showed no difference [15]. In order to confirm our previous *in vitro* data, we performed a Ki67 staining on all the tumors at 8 weeks. There was no noticeable difference in Ki67 staining between the pcmv and miR-145 tumors (Figure 4D). This confirmed that the tumor size difference was not due to the cell line proliferation rate. As it is well established that the tumor-microenvironment can affect the tumor growth, it is possible that the SK-GT-4 miR-145 cells have the potential to modulate the microenvironment to stimulate growth. Previous reports of the role of miR-145 on fibroblasts and endothelial cells demonstrated that expression of miR-145 results in the transformation of fibroblasts to myofibroblasts and the stromal expression of miR-145 resulted in increased angiogenesis, both of which are known to increase and maintain the tumor growth [19, 20]. In order to verify this hypothesis, we measured the amount of miR-145 released by the both cell lines. The SK-GT-4 miR-145 cell line expressed and secreted around a 1000 fold more miR-145 than the SK-GT-4 pcmv cell line (Figure 5A). Expression of mouse CD31 in our SK-GT-4 tumors was evaluated but there were no clear differences in CD31 staining between the pcmv and miR-145 tumors (Figure 5B). Therefore, it is unlikely that the size difference between pcmv and miR-145 tumors is due to angiogenesis.

Micrometastasis was found in the lung of only one of 25 mice on H & E, (SK-GT-4 mir-145) (Figure 5C). This was a surprising result since the SK-GT-4 displayed a high invasive potential *in vitro*. However, previous reports described metastasis occurring around the 40 week mark, which could explain the lack of metastasis observed in the lungs. Previously, our collaborators used FISH technique to detect transplanted mesenchymal stem cells in pig lungs [16]. This technique allows detection of single cancer cells. When this technique was used, cancer cells were detected in 10 out of 13 mice lungs in the SK-GT-4 miR-145 xenografts but in only 4 out 12 mice lungs for the SK-GT-4 pcmv xenografts (Figure 5D). To our knowledge, this is the first report, which describes detection of lung metastasis from an EAC xenograft flank model using NOD/SCID mice. After previously establishing the miR-145 role in EAC *in vitro*, it appears that miR-145 expression can also lead to a higher ability to metastasize *in vivo* and also form bigger tumors in a small animal model. This higher rate of metastasis in miR-145 tumors could be explained by the ability of miR-145 to enhance α5β1and αvβ3 integrin expression, which leads to a higher resistance to anoikis and potential for invasion.

## DISCUSSION

We previously demonstrated that miR-145 expression in EAC cell lines promotes cell invasion, resistance to anoikis and increased their ability to bind fibronectin. In this report, we investigated the signaling pathways responsible for the oncogenic abilities of miR-145 in EAC. We discovered that SK-GT-4 miR-145 cells expressed higher level of integrins α5 and β3 compared to the SK-GT-4 pcmv cells. Since miRNAs can only repress expression of their target, we looked for an integrin α5 and β3 repressor that was a known miR-145 target. c-Myc is a known repressor of both integrin subunits and also a miR-145 target. We demonstrated that the expression of c-Myc and integrins α5 and β3 were inversely correlated and that we could reproduce the miR-145 effects on cell adhesion to fibronectin and anoikis resistance in SK-GT-4 pcmv cells by knock down of c-Myc expression. Also, we showed that integrin α5 mediates the enhancement of fibronectin adhesion observed in the SK-GT-4 miR-145 cells whereas integrin β3 controls anoikis resistance and the higher invasion potential of SK-GT-4 miR-145 cells. Finally, we showed that miR-145 expression in SK-GT-4 led to bigger, more aggressive tumors in NOD/SCID mice and also, increased metastasis to the lungs.

An interesting finding with our tumor model was the fact that SK-GT-4 miR-145 cells created bigger tumors than the SK-GT-4 pcmv cells. This is surprising because we previously described that there was no differences in cell proliferation between the cell lines. Therefore, it is possible that the miR-145 tumors contain more myofibroblasts than the pcmv tumors, which could potentially explain the difference in tumor size and this would highlight a dual oncogenic ability of miR-145 by affecting not only the cancer cells but also the tumor microenvironment.

To our knowledge, this is the first report showing spontaneous metastasis in NOD/SCID mice with an EAC cell line subcutaneous flank model. A recent report showed spontaneous metastasis with FLO-1 cell line in NSG mice, but no metastases were reported in the NOD/SCID mice [21]. After 8 weeks, we could only detect one metastasis using H&E in one of 25 mice (with SK-GT-4 miR-145 cells), but by using the FISH technique, we could detect twice as many tumor cells in the mice lungs of mi-145 group compared to the pcmv group. Despite the correlation between this result and the enhanced invasion ability of the miR-145 cells *in vitro*, it is possible that the difference in metastasis in the lungs we observed could be due to enhanced cell proliferation of miR-145 cells *in vivo* since the miR-145 tumors are twice as big as the pcmv tumors at the time of sacrifice. When we assessed the cell proliferation rate in the tumors, we observed no difference between the two groups, which matched our *in vitro* cell proliferation data. Therefore, it is unlikely that cell proliferation contributes to the difference in metastasis potential. Our *in vitro* data suggested that miR-145 only acts on invasion potential and metastasis related events, therefore the size difference may come from an indirect effect of miR-145 on the tumor microenvironment as mentioned previously. Therefore, the miR-145 effect on metastasis *in vivo* may be partially explained by the results we obtained *in vitro*. The SK-GT-4 cell line has high invasive potential and robust clonogenic ability *in vitro* so it was surprising that the metastatic potential of this cell line is so poor. In the clinical situation, esophageal adenocarcinoma is highly metastatic [3]. One possible explanation of this difference could be that the architecture of the esophagus and draining lymphatic system facilitates the dispersion of cells from the primary tumor.

This report further highlights a new potential way to identify metastases in animal models. The use of FISH combined with the ALU sequence allowed us to specifically identify individual human cancer cells, and therefore we could develop a precise time lapse of the dispersion of cancer cells from the primary tumor in an animal model.

In this report, we showed that expression of miR-145 in the SK-GT-4 cell line resulted in the formation of bigger tumors in NOD/SCID mice and a higher ability to metastasize to the lungs, validating our previous *in vitro* data. It appears that the oncogenic ability of miR-145 requires a downregulation of the miR-145 target, c-Myc, which results in increased expression of integrins α5 and β3. Integrin α5 controls the adhesion to fibronectin whereas integrin β3 partially controls anoikis resistance and cell invasion of SK-GT-4 cells.

## MATERIALS AND METHODS

### Cell lines

OE33 and SK-GT-4 cell lines were purchased from the European Collection of Cell Cultures (ECACC, UK). All cell lines were cultured in RPMI 1640 with 10% FCS and 1mM L-Glutamine and Penicillin/Streptomycin at 37°C and 5% CO_2_. The SK-GT-4 pcmv and miR-145 cell lines were established as previously described [15].

### Development of xenograft tumors from human esophageal adenocarcinoma cell lines in NOD/SCID mice

Human esophageal carcinoma SK-GT-4 parental cells (SK-GT-4 pcmv) and miRNA 145-transfected SK-GT-4 miR145 cells (5×10^6^/100μl PBS) were injected subcutaneously (sc) into the right flank of the NOD/SCID mice. Tumor size was measured when tumor nodules became palpable, approximately 3-5 days after tumor cell injection. The two maximal perpendicular diameters of the tumors were measured once weekly with a caliper. Tumor volume was calculated according to a formula V=*ab*^2^π/6, where *a* and *b* represents the longest perpendicular diameters, respectively. All procedures followed the animal care regulations of University Health Network after approval by the Research Ethics Board. Mice were sacrificed at 8 weeks after tumor cell injection, and the lung, liver, and tumor tissue were collected.

### Paraffin embedding and H&E

Tumors, lungs and livers were fixed and embedded in paraffin blocks. Slides from each block was made and stained with Hematoxylin and Eosin following standard protocol. The presence of micro-metastasis in lungs and liver was assessed by light microscopy. All the samples were processed by the Pathology Research Program Laboratory at UHN.

### Fluorescence *in situ* hybridization

Tracking of the SK-GT-4 cells in the lung was performed using fluorescence *in situ* hybridization (FISH), as described elsewhere [16]. Briefly, paraffin slides from tumor biopsies and at the end of the experiments were hybridized with an ALU DNA probe (Cat. No. Q151P.0100; Life Technologies).

### siRNA transfection

The establishment of SK-GT-4 pcmv and miR-145 cell lines was achieved as previously described [15]. For siRNA transfection, cells were plated (10^5^/well) in a 6 well plate 24 h prior to transfection. 100 nmol siRNA in 200 μl of Jet–Prime buffer (Polyplus, France) was mixed with 2μl of Jet Prime transfection reagent (Polyplus, France) and allowed to incubate 10 mins at room temperature. 200 μl of this mixture was then added to the cells in 1.8 ml of RPMI 1640 with 10%FCS and incubated 48h. After 48h, the medium was changed to RPMI with 10%FCS. The efficacy of the siRNA transfection was verified 72h after transfection.

### Cell proliferation

Cells were plated at 5×10^4^/well in a 6 well plate. After 72h of incubation, cells were trypsonized and resuspended in 1ml of RPMI 1640 with 10%FCS. Cells were then counted with a haemocytometer using the trypan blue exclusion method.

### Western blotting

Western blotting was used to measure integrin subunits and c-Myc expression. Cells were washed and then lysed using RIPA buffer. Proteins were then separated on an SDS-polyacrylamide gel (Bio-Rad) and then transferred to a PVDF membrane using a Semi Dry Blotting system (Bio-Rad). Membranes were then incubated overnight at 4°C with primary antibodies. The membranes were then washed and incubated with secondary HRP antibody for 1h at room temperature. The membranes were then incubated with Super Signal West Pico chemiluminescent substrate kit (Thermo Scientific) and processed using a Bio-Rad developer. PARP ((46D11) Rabbit mAb #9532, dilution 1:10000), Beta-Actin Rabbit mAb #4967, dilution 1:10000), Cleaved Caspase 3 (Asp175) (5A1E) Rabbit mAb #96, dilution 1:1000, Cleaved Caspase 8 Rabbit mAb, dilution 1:1000, integrin α5 Rabbit mAb, dilution 1:3000, integrin β1 Rabbit mAb, dilution 1:10000, integrin β3 Rabbit mAb, dilution 1:1000, integrin β4 Rabbit mAb, dilution 1:1000, integrin αv Rabbit mAb, dilution 1:1000, c-Myc mAb, dilution 1:3000 and anti-rabbit HRP ((#7074) dilution 1:5000) antibodies were purchased from Cell Signaling.

### Cell adhesion assay

2×10^5^ cells were plated in a well (6 well plate) pre-coated with 15 μg/ml Fibronectin. Cells were incubated for 30 mins at 37°C with 5% CO_2_. Detached cells were then counted as described previously. For the blocking antibody experiments, cells were pre-incubated at 4°C for 1h with 10 ug/ml of either isotype antibody or the antagonist antibody.

### Cell invasion assay

Cell invasion was assessed using CytoSelect Cell invasion Assay, Basement Membrane (Cell Biolabs, USA) following manufacturer's protocol. 15×10^4^ cells in RPMI were incubated 48h on top of RPMI 1640 with 10%FCS. The invasion rate was determined by spectrophotometer as described by the protocol. For the blocking antibody experiments, cells were pre-incubated at 4°C for 1h with 10 ug/ml of either isotype antibody or the antagonist antibody.

### Anoikis assay

10^5^ cells were plated on Low Adhesion plates (Corning) and cultured for 72h at 37°C. After 72h, cells were harvested and lysed with RIPA buffer. The level of anoikis was assessed using Western Blotting by looking at the cleavage of PARP, caspase 8 and caspase 3. For the blocking antibody experiments, cells were pre-incubated at 4°C for 1h with 10 ug/ml of either isotype antibody or the antagonist antibody.

### Animal model

Control cells (pcmv) and miR-145 expressing EAC cells (SK-GT-4) were injected in the flank of mice. The route of flank injection was chosen over tail vein injection as this was considered to be more representative of the true metastatic process. At sacrifice, mice lungs and liver were evaluated for the presence of human EAC cells using immunohistochemistry and fluorescence *in situ* hybridization.

### RT-PCR

Cell lysates from SK-GT-4 pcmv and miR-145 were treated as previously described [15].

The condition medium was obtained from 10 ^6^ cells cultured 24h. The miRNA extraction from the condition medium of SK-GT-4 cells was performed using Exiquon miRNA kit. After miRNA extraction, the condition medium samples were analyzed as previously described [15].

### Statistical analysis

All data are presented as the mean ±SE from at least two independent experiments. Each sample was done in triplicate. Statistical analysis was performed by Student's t test or Mann Whitney test. Statistical significance is shown as ^*^, meaning p<0.05.

## SUPPLEMENTARY MATERIALS FIGURE



## Abbreviations

CLL: Chronic Lymphocytic Leukemia
EAC: Esophageal Adenocarcinoma
FCS: Foetal Calf Serum
HRP: Horseradish Peroxidase
PVDF: Polyvinylidene
