# Supplementary material for: miR-145 expression enhances integrin expression in SK-GT-4 cell line by down-regulating c-Myc expression
Source: Oncotarget. 2018 Mar 8;9(20):15198–207. doi: 10.18632/oncotarget.24613 (PMC5880596; doi:10.18632/oncotarget.24613)
Supplement: Supplementary file 1 [file oncotarget-09-15198-s001.pdf]

miR-145 expression enhances integrin expression in SK-GT-4 cell line by down-regulating c-Myc expression

SUPPLEMENTARY MATERIALS

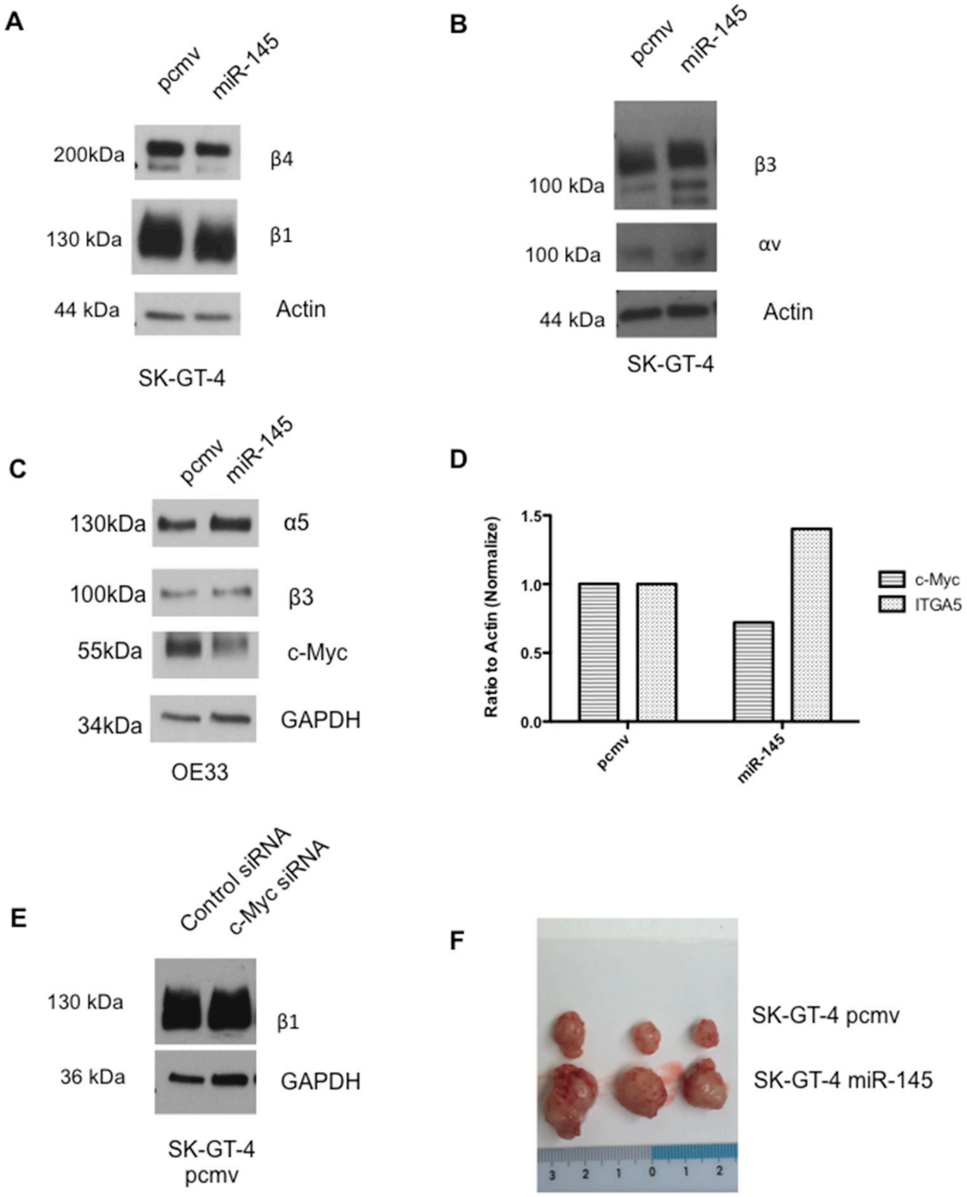

Supplementary Figure 1: (A) Western blot looking at the expression of integrins β1, β3 in SK-GT-4 pcmv and miR-145. **(B)** Measurement by densitometry of the c-Myc and ITGA5 bands in SK-GT-4 pcmv and miR-145 cells. This data is representing the data showed in Figure 1A. **(C)** Western Blot looking at the expression of integrins αv, β3 in SK-GT-4 pcmv and miR-145. **(D)** Western Blot looking at the expression of integrins α5, β3 and c-Myc in OE33 pcmv and miR-145. **(E)** Western Blot analysis of integrin β1 after siRNA transfection in SK-GT-4 pcmv cells. **(F)** Pictures of the SK-GT-4 tumors after 8 weeks.
